# Ultrasound-guided lateral pterygoid muscle injection for inferior alveolar nerve block in sagittal split ramus osteotomy: a three-case series

**DOI:** 10.1186/s40981-025-00799-4

**Published:** 2025-06-16

**Authors:** Keisuke Nakazawa, Ryota Tsukui, Yoshio Ohyama, Yoshinori Inaba, Junko Tamari, Takahiro Suzuki

**Affiliations:** 1 Department of Anaesthesiology, Nihon University School of Medicine, 30-1 Kamimachi, Ooyaguchi, Itabashi-Ku, Tokyo, 173-8610 Japan; 2 Department of Anaesthesiology, Shizuoka City Shizuoka Hospital, Shizuoka, Japan; 3 Department of Oral and Maxillofacial Surgery, Shizuoka City Shizuoka Hospital, Shizuoka, Japan

**Keywords:** Regional anesthesia, Inferior alveolar nerve block, Ultrasound guidance, Lateral pterygoid muscle, Orthognathic surgery

## Abstract

**Background:**

Sagittal split ramus osteotomy is often associated with significant postoperative pain. Intraoral inferior alveolar nerve blocks have variable success rates and higher risks of vascular complications, while ultrasound-guided approaches to the pterygomandibular space require precise needle placement in a narrow anatomical space. We present a novel perioperative application of ultrasound-guided lateral pterygoid muscle injection for regional anesthesia.

**Case presentations:**

Three female patients underwent bilateral sagittal split ramus osteotomy under general anesthesia. After anesthesia induction, ultrasound-guided lateral pterygoid muscle injections were performed using 10 mL of 0.25% levobupivacaine. All patients demonstrated excellent postoperative pain control (numerical rating scale score ≤ 2) with minimal analgesic requirements and no complications.

**Conclusion:**

This novel lateral pterygoid muscle injection technique for perioperative analgesia demonstrates promising clinical efficacy through a simplified ultrasound-guided approach, providing effective opioid-free postoperative pain management for sagittal split ramus osteotomy.

## Background

Sagittal split ramus osteotomy (SSRO) typically results in significant postoperative pain requiring multimodal analgesia, often including opioid-based intravenous patient-controlled analgesia (IV-PCA). Prior to the introduction of ultrasound-guided nerve blocks at our institution, we managed all cases with opioid-based anesthesia and postoperative IV-PCA (fentanyl 20 µg/ml/h, bolus dose 20 µg, lock-out time 10 min). The management of postoperative analgesia, reliant on opioids and the manifestation of postoperative nausea and vomiting (PONV) and the presence of severe pain unresponsive to opioid treatment has considerably exacerbated the complexities associated with postoperative care administration.

Traditional intraoral inferior alveolar nerve blocks targeting the pterygomandibular space have been the standard approach for decades. However, they are associated with variable success rates, risks of vascular complications, and cannot be performed in patients with limited mouth opening. More recently, ultrasound-guided inferior alveolar nerve block (UG-IANB) has been introduced as an alternative approach for delivering local anesthetic to the pterygomandibular space (PMS) [1, 2]. While UG-IANB offers improved precision compared to the intraoral approach, it still presents certain limitations: it requires accurate needle placement within a narrow anatomical space containing important vasculature, and visualization challenges may arise in patients with obesity or limited mouth opening, potentially compromising block success.

Our novel approach targeting the lateral pterygoid muscle (LPM) aims to simplify the procedure while maintaining clinical efficacy, particularly in challenging patients. LPM provides clear ultrasonographic visualization and is a reliable anatomical landmark even with minimal mouth opening. Notably, lateral pterygoid muscle injection (LPMI) is commonly used for chronic pain conditions such as temporomandibular disorders and mandibular region pain. However, to our knowledge, there are no reports of its use in the perioperative setting for maxillofacial surgery.

In this case series, we describe three patients undergoing bilateral SSRO who received this novel ultrasound-guided LPM injection technique for perioperative pain management.

## Case presentations

After obtaining institutional ethics committee approval (approval number: 24–103) and written informed consent, we performed our novel technique on three female patients (aged 18–33 years) scheduled for bilateral SSRO.

1. Pain assessment protocol: Patient pain was assessed using an 11-point numerical rating scale (NRS 0–10), with evaluations conducted by high-care unit nurses four times daily (8:00, 12:00, 16:00, and 20:00) for 24 h postoperatively. Both pain at rest and pain during mouth opening were assessed. Patients were instructed to use the nurse call system if they experienced severe breakthrough pain at any time, ensuring flexible administration of analgesics as needed. All pain assessments were documented in the electronic medical record system, and this information was reviewed during routine postoperative visits by the anesthesiologists.
2. Standard treatment protocol: Prior to implementing this ultrasound-guided LPM technique, our standard SSRO protocol included general anesthesia with remifentanil maintenance followed by fentanyl (5–8 µg/kg total) during the final 2 h of surgery and postoperative IV-PCA using a 100-ml bottle with fentanyl (20 µg/ml/h, bolus dose 20 µg, lock-out time 10 min). This IV-PCA was typically continued for 48 h to a maximum of 3 days. It was primarily implemented to manage the most severe pain during the first 24 h postoperatively. For patients who received the ultrasound-guided LPM technique, anesthesia was maintained with remifentanil only, without additional fentanyl administration during surgery, and IV-PCA was not routinely used postoperatively. In all cases, acetaminophen (15 mg/kg) was administered slowly before emergence from general anesthesia. If patients showed signs of poor pain control in the operating room upon emergence (NRS > 5 or clinical judgment by the anesthesiologist or oral-maxillofacial surgeon), IV-PCA fentanyl was promptly initiated.
3. Postoperative analgesia protocol: For the patients in this case series, our protocol was as follows: When pain at rest or during mouth opening was ≥ 5 on the NRS or if the patient strongly requested pain medication, loxoprofen 60 mg was administered as needed (minimum 6-h intervals). If loxoprofen was required more than twice, scheduled acetaminophen 1500 mg daily in 3 divided doses (every 8 h) was considered. If pain persisted with NRS ≥ 5 despite the administration of loxoprofen and acetaminophen, the anesthesiologist and oral-maxillofacial surgeon would collaborate to consider implementing IV-PCA (fentanyl).
4. Specific case details: In all three cases, anesthesia was induced with remifentanil (0.5 µg/kg/min) and propofol using a target-controlled infusion (TCI), followed by rocuronium for nasotracheal intubation. Anesthesia was maintained with a propofol TCI adjusted according to BIS monitoring and a remifentanil dose of 0.15 µg/kg/min. No additional opioids, such as fentanyl or morphine, were administered during the perioperative period.

**Fig. 1:**
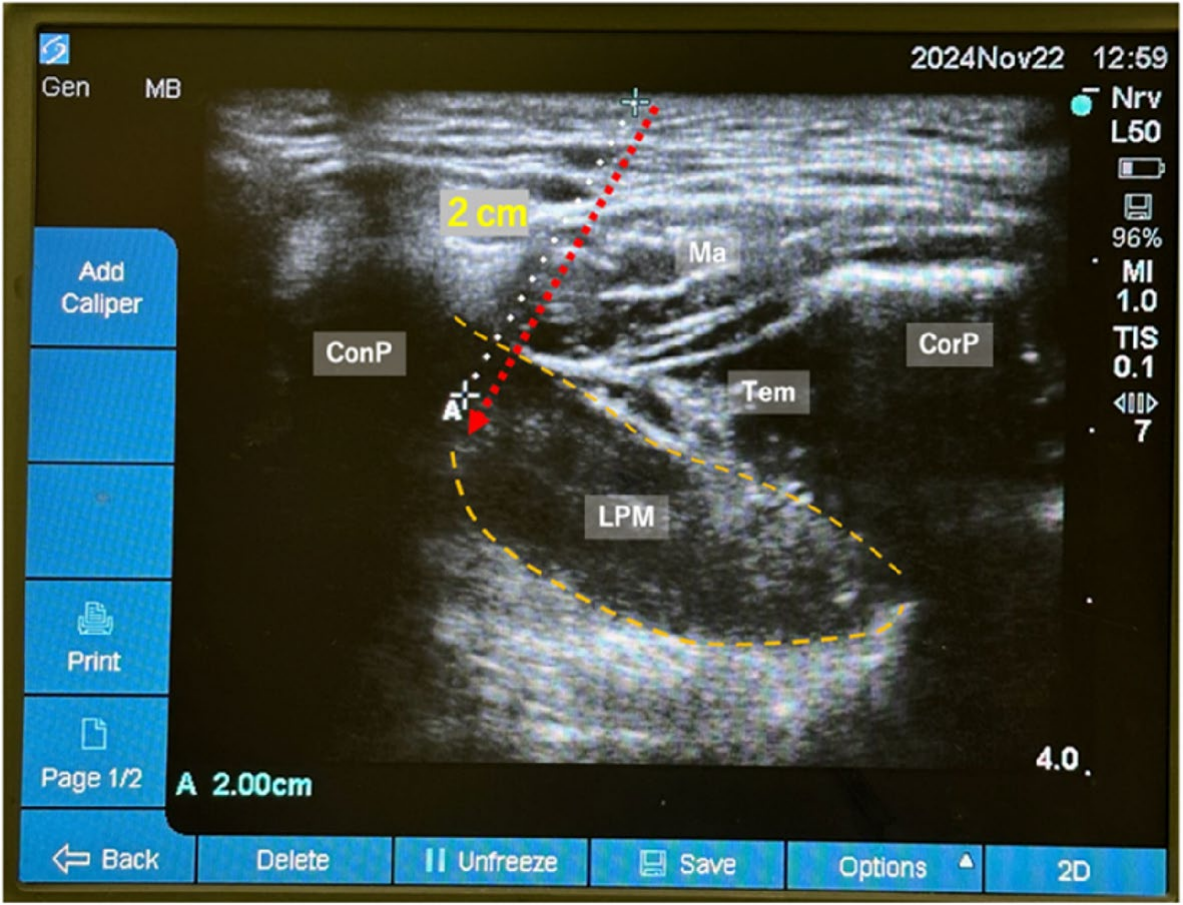
Sonographic anatomy for the lateral pterygoid muscle approach. The yellow dotted line outlines the lateral pterygoid muscle. The red dotted arrow indicates the estimated needle path. The white dotted line shows the distance (2 cm) from the probe surface to the condylar process. Note the relationship between the condylar process (ConP), coronoid process (CorP), masseter (Ma), temporalis muscle (Tem), and lateral pterygoid muscle (LPM)

**Fig. 2:**
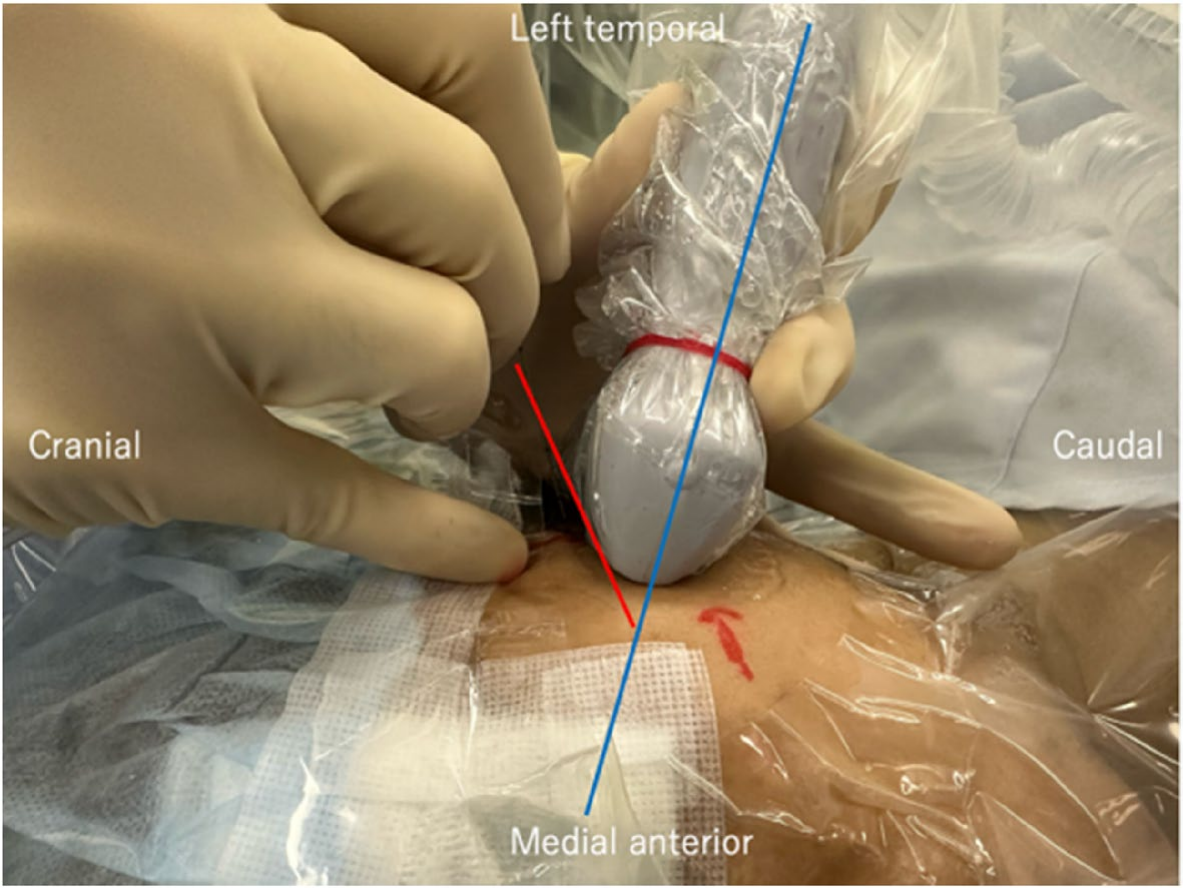
Diagram of needle orientation showing the 45–60° approach angle relative to the ultrasound probe positioned below the zygomatic arch

**Fig. 3:**
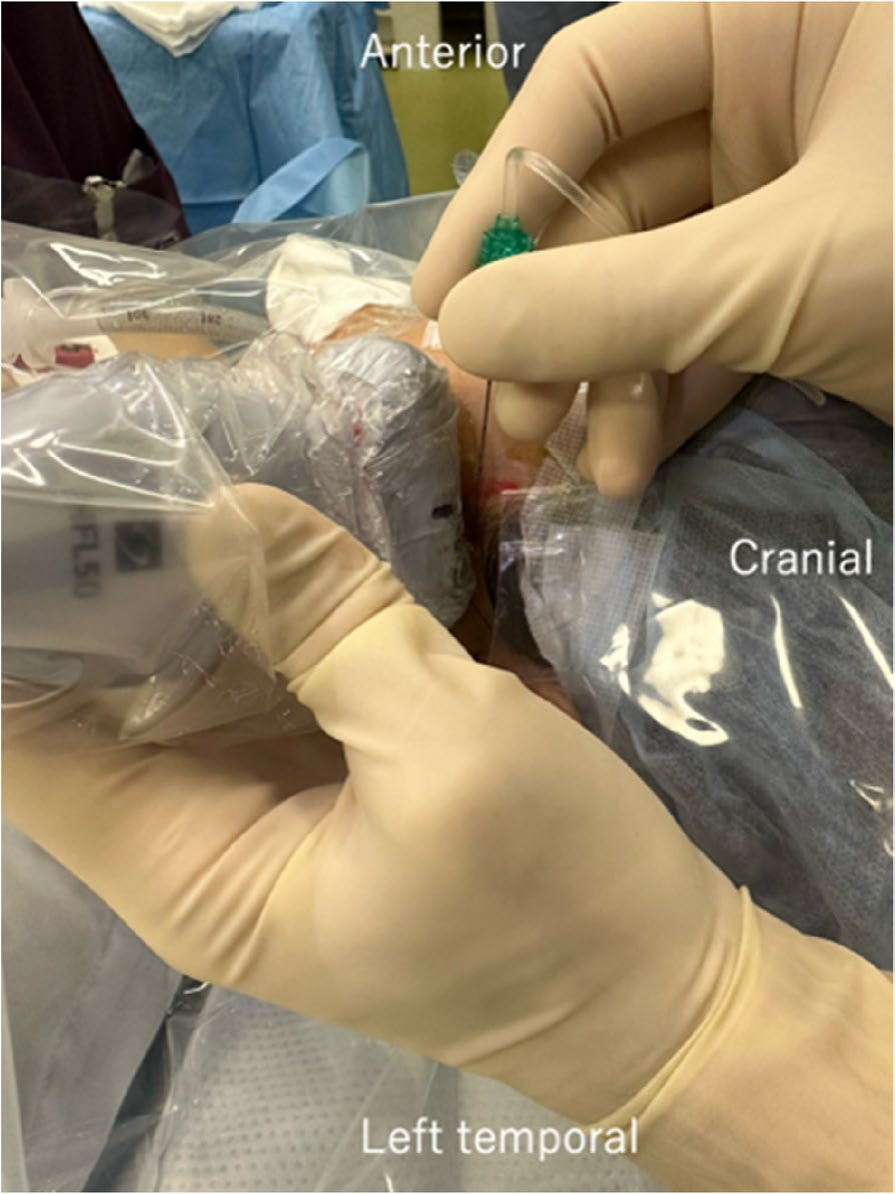
Clinical photograph demonstrating probe position and needle insertion angle in the out-of-plane approach

The nerve block (LPMI) was performed immediately after general anesthesia induction, before surgical incision, under ultrasound guidance, with a 6–15 MHz linear probe positioned parallel to the zygomatic arch. The key anatomical structures identified included the masseter, coronoid process, LPM, and temporalis muscle (Fig. 1). After the target anatomy was visualized, we repeatedly tilted the ultrasound device in the superior-inferior direction to identify the pulsation of the maxillary artery between the lateral pterygoid muscle and the masseter muscle. Color Doppler was used to confirm the presence or absence of vessels further. To avoid vascular complications, we fixed the linear probe face to the skin and repeatedly tilted it, confirming that no arteries were present in the needle path. We also identified the superficial temporal artery to avoid inadvertent puncture. The movement of tissues as the blunt needle advanced and the acoustic shadow easily confirmed the needle tip position. Using an out-of-plane technique, the needle was inserted cranio-caudally at a 45–60° angle and directed 10–20° posteriorly (Figs. 2 and 3).

**Fig. 4:**
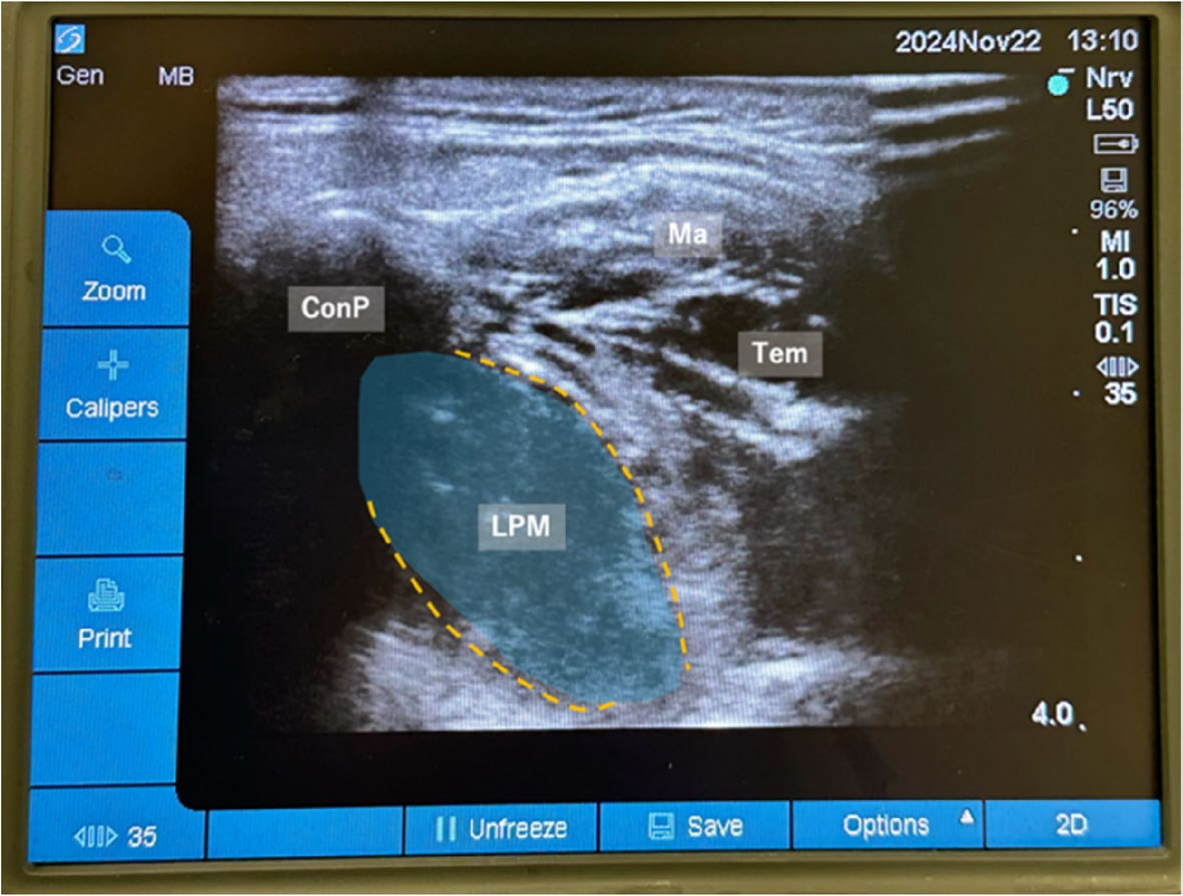
Ultrasound image showing local anesthetic spread (blue shading) within the lateral pterygoid muscle (yellow dotted outline) after its injection. Note the relationship between the condylar process (ConP), coronoid process (CorP), masseter (Ma), temporalis muscle (Tem), and lateral pterygoid muscle (LPM)

After a negative aspiration test, 10 mL of 0.25% levobupivacaine (a total of 20 mL for both sides) was injected while ultrasonographically observing muscle expansion (Fig. 4). In all cases, acetaminophen (15 mg/kg) was administered at surgery completion. For PONV prevention, dexamethasone 3.3 mg IV and ondansetron 4 mg IV were administered.

In the first patient (33 years, 167 cm, 45 kg), pain was maintained at a numerical rating scale (NRS) score of ≤ 2 throughout the entire 24-h observation period without requiring any rescue analgesics during or after her 179-min procedure, demonstrating effective pain control from the LPMI technique alone. The second patient (25 years, 157 cm, 56 kg) underwent a 240-min procedure and required one dose of loxoprofen 60 mg at 4 h postoperatively when her NRS score reached 5, after which pain was maintained at an NRS score of ≤ 2. The third patient (18 years, 160 cm, 62.9 kg) had a 333-min procedure and received one rescue dose of loxoprofen 60 mg at 2 h postoperatively when the NRS score reached 5, after which the pain remained well-controlled (NRS score ≤ 2). The pain was effectively managed with loxoprofen alone, and no additional analgesics, such as acetaminophen or opioids, were required during the 24-h observation period in the high-care unit. All patients maintained stable NRS scores of ≤ 2 throughout this period. Additionally, all patients demonstrated stable hemodynamics throughout, and no postoperative complications such as nausea, vomiting, swelling of the surgical site, or trismus were observed. Postoperative creatine kinase (CK) levels measured on POD 0 were 89 U/L for the first patient, 56 U/L for the second patient, and 86 U/L for the third patient, all within normal reference ranges (76–153 U/L).

After the initial 24-h acute observation period in the high-care unit, all patients were transferred to the general ward, where scheduled dexamethasone 3.3 mg twice daily was administered for 3 days. While all three patients reported no pain at rest (NRS 0), they experienced pain during mouth opening (NRS > 5), necessitating additional analgesics that successfully maintained pain control (NRS < 2). Notably, no opioids were prescribed beyond the first 24 h for any patient.

The first patient required loxoprofen until POD 4, with acetaminophen added to POD 1. The second patient was prescribed loxoprofen on POD 1 only. The third patient continued loxoprofen until POD 3. Importantly, follow-up examinations revealed no evidence of late trismus in any of the three patients through discharge dates. No patients underwent specific imaging studies (ultrasound or MRI) of the lateral pterygoid muscle during this period, as they showed no clinical signs suggestive of significant muscle injury that would warrant such investigations. All patients were discharged without complications according to the scheduled clinical pathway (the first patient on POD 10, the second patient on POD 8, and the third patient on POD 10) and began their regular outpatient follow-up visits and rehabilitation of jaw mobility and occlusal function.

## Discussion

Ultrasound-guided inferior alveolar nerve block (UG-IANB) is a mandibular analgesic procedure in which local anesthetic is injected into the pterygomandibular space (PMS). Kumita et al. investigated the effectiveness of UG-IANB in delivering local anesthetic to the PMS and its ability to stain the mandibular nerve trunk and its branches, using a cadaveric model with 4 Thiel-embalmed cadavers and 8 UG-IANB procedures.

The study involved performing UG-IANB on the bilateral faces of 4 Thiel-embalmed cadavers, where a needle was advanced to the PMS under ultrasound guidance, and 5 mL of dye was injected to assess the spread of the injectate. The findings indicated that while UG-IANB successfully stained the inferior alveolar nerve (IAN), lingual nerve (LN), and buccal nerve (BNs), it did not reach the mandibular nerve (MN) or auriculotemporal nerve (ATN) located outside the PMS, suggesting that UG-IANB can provide sufficient analgesia for mandibular surgeries without affecting these nerves. Several studies have reported the clinical superiority of UG-IANB over the intra-oral IANB technique in which a needle is advanced blindly to the PMS through the oral mucosa [1].

While intra-oral IANB techniques have reported success rates of 75–85% [3, 4], they are reportedly associated with an incidence of blood aspiration of 2.9–22% [5]. Furthermore, this approach cannot be used in patients with limited mouth opening. Kojima et al. reported a case in which UG-IANB was successful in a patient with a mouth-opening disorder, resulting in improved mouth opening [6].

Our inferior alveolar nerve block technique for SSRO represents a significant modification from ultrasound-guided IANB. It targets the lateral pterygoid muscle rather than attempting precise placement of the local anesthetic within the pterygomandibular space (PMS).

UG-IANB targeting the PMS is a more superficial block compared to nerve blocks targeting the lateral pterygoid plate [7]. While following similar ultrasound-guided techniques [2, 7], our approach offers several potential advantages through improved ultrasonographic visualization of the LPM, which might reduce procedure time and technical complexity while potentially decreasing the risk of vascular complications.

The reliability of our technique might be partly explained by the anatomical consistency of the LPM as an ultrasonographic landmark. Chang et al. demonstrated that the LPM provides clear ultrasonographic visualization and is a reliable anatomical reference point for trigeminal nerve blocks to advance the needle to the pterygopalatine fossa in this region using a curvilinear probe [8]. Our method requires needle advancement of only about 20 mm from the skin surface and does not require a curvilinear probe. While we use a cross-sectional approach, the shallow needle path allows easy visualization of the needle tip, reducing the risk of inadvertent puncture of the superficial temporal artery running along the skin surface and the maxillary artery running along the surface of the LPM.

Our LPMI technique is simple, involving only advancing the needle tip into the lateral pterygoid muscle and injecting a local anesthetic. This method may be particularly effective in patients with complex LPM visualization, such as those with limited mouth opening or obesity. Typically, LPM visualization improves when the mouth is opened, as the coronoid process (CorP) moves inferiorly, widening the space for needle advancement and clarifying anatomical recognition. Our LPMI technique allows for the advancement of the needle into the LPM through the limited space between the coronoid process (CorP) and condylar process (ConP) using a cross-sectional approach, even without mouth opening. We can ensure accurate intramuscular injection by measuring the distance to the muscle before puncture.

Our findings align with those of Kumar et al. [9], who demonstrated effective pain control using ultrasound-guided trigeminal nerve blocks targeting the pterygopalatine fossa (PPF). The close anatomical relationship between the LPM and PPF, separated only by the lateral pterygoid plate, suggests that the local anesthetic might effectively spread to the relevant nerve branches through this indirect approach.

Our LPMI technique showed excellent postoperative pain control in all three cases. It is particularly noteworthy that our first patient (33 years, 179-min procedure) maintained excellent pain control (NRS ≤ 2) throughout the entire 24-h observation period without requiring any rescue analgesics, suggesting that the LPMI technique alone provided sufficient analgesia in this case. Even our third patient, who underwent the longest procedure (333 min, 18 years) and thus potentially experienced greater surgical trauma, required only a single dose of loxoprofen 60 mg for rescue analgesia when the NRS score reached 5, after which pain was maintained at NRS ≤ 2. The second patient (25 years, 240-min procedure) similarly required only one dose of loxoprofen. This consistent pattern of minimal analgesic requirements across varying surgical durations indicates the potential value of this technique as a cornerstone of opioid-sparing analgesic strategies for SSRO.

This novel ultrasound-guided lateral pterygoid muscle injection technique demonstrated effective pain control primarily during the first 24 h after SSRO, which is typically the most painful period following this procedure. Throughout this critical initial recovery phase, all three patients remained opioid-free, with pain successfully managed using only non-opioid analgesics when needed.

As expected, when the effect of the local anesthetic diminished after the 24-h observation period, patients experienced increased pain during mouth opening, requiring additional non-opioid analgesics. However, even beyond the 24-h mark, none of the patients required opioid administration. Instead, a multimodal analgesic approach consisting of scheduled dexamethasone as an anti-inflammatory agent, acetaminophen, and loxoprofen was sufficient to maintain adequate pain control, suggesting that the initial nerve block set the foundation for effective ongoing pain management without opioids throughout the recovery period. All patients were discharged without complications: the 33-year-old patient on POD 10, the 25-year-old patient on POD 8, and the 18-year-old patient on POD 10. The uneventful recovery and timely discharge further support the safety and efficacy of this technique as part of a comprehensive perioperative management approach.

Although these case reports include only three patients, and the comparison between cases has limited significance, the effective pain control without IV-PCA is particularly noteworthy considering that many female SSRO patients at our institute discontinue IV-PCA because of PONV. This suggests that our LPM injection technique might enable effective opioid-free postoperative pain management for SSRO procedures through a multimodal approach using only conventional non-opioid analgesics.

Regarding the analgesic efficacy of our technique, we acknowledge the potential contribution of dexamethasone (3.3 mg), administered intraoperatively for PONV prevention, to the prolonged analgesic effect. Systemic steroid administration has been shown to extend nerve block duration and reduce postoperative pain and swelling. This effect likely complemented our LPMI technique as part of the multimodal analgesic approach. The consistently well-controlled postoperative pain observed across all three cases suggests the efficacy of this approach. Our LPM injection technique demonstrates promising clinical efficacy through a simplified ultrasound-guided approach. This technique provides adequate opioid-free postoperative analgesia for SSRO while offering technical advantages through clear anatomical visualization.

We acknowledge several limitations in this initial case series. While our technique was successful in all three patients, the potential influence of anatomical variations in the mandibular nerve distribution might affect the consistency of blocks in larger patient populations [10]. In our LPMI approach, we injected 10 mL of 0.25% levobupivacaine into the muscle on each side. We should consider the potential toxicity of local anesthetics to muscle tissue. Anatomically, the LPM typically consists of two heads: the superior (SLPM) and inferior (ILPM) heads. However, variations such as additional medial and middle heads have been reported, along with unique-headed configurations [11].

Our technique of injecting 10 mL of local anesthetic into the lateral pterygoid muscle (LPM) raises important considerations regarding volume-to-muscle ratio. Balcioglu et al. demonstrated that the LPM has a relatively small volume, with the superior head measuring approximately 2.9 cm^3^ and the inferior head about 5.5 cm^3^ on lateral sides [12]. With our 10 mL injection significantly exceeding the anatomical muscle volume, the anesthetic solution likely spreads beyond the muscle tissue itself.

This volumetric excess may explain the comprehensive analgesic effect observed in our cases, and the solution likely infiltrates surrounding anatomical structures relevant to pain pathways. However, this spread also raises considerations regarding potential complications. The LPM’s proximity to important neurovascular structures, including the maxillary artery, necessitates careful consideration of injection volumes to avoid inadvertent vascular uptake and systemic toxicity.

We must also consider potential significant muscle injury from local anesthetic injection into the muscle itself. Levobupivacaine causes less significant muscle injury and inflammatory response compared to bupivacaine. Studies show that levobupivacaine results in lower levels of pro-inflammatory cytokines such as TNF-α, IL-1, and IL-6 and fewer apoptotic cells in muscle tissue [13]. Electron microscopic examination has confirmed that all three commonly used local anesthetics (bupivacaine, ropivacaine, and levobupivacaine) cause similar qualitative significant muscle injury. However, levobupivacaine produces quantitatively less myotoxic effects at both cellular and tissue levels [14]. Significant muscle injury peaks around the fourth day post-injection for both anesthetics, but levobupivacaine shows less persistent damage and faster recovery [15].

Here, we explicitly acknowledge that local anesthetic exceeding the anatomical volume of a muscle may increase the risk of myotoxicity, and higher volumes may cause increased tissue pressure, mechanical disruption, and prolonged exposure of muscle fibers to local anesthetic agents. Despite an extensive literature review, we found a significant knowledge gap regarding studies that specifically quantify the relationship between volume-to-muscle ratio and myotoxic risk in humans or animal models. However, a systematic review of local anesthetic-induced myotoxicity reported that recovery time in human muscles can range from 4 days to 1 year, with complete recovery observed in only 38% of patients, a concerning statistic that warrants careful consideration when using high-volume injections [16].

There is also a documented risk of trismus following excessive volumes of local anesthetic solution deposited into bounded regions of the infratemporal fossa, which contains the pterygoid muscles. This trismus may occur due to tissue expansion, hematoma formation, or direct trauma to the muscles, as documented in a recent case report where severe trismus (mouth opening limited to 18 mm) following an inferior alveolar nerve block required 50 days of comprehensive treatment before full recovery was achieved [17]. Additionally, review articles have indicated that myotoxicity tends to occur with high doses or concentrations of local anesthetics and/or high injection pressure [18, 19].

Several preventive measures may be considered to minimize these risks: using a minimal adequate volume of local anesthetic, performing slow injections to avoid tissue trauma, and considering perioperative corticosteroid administration. Studies have shown that submucosal dexamethasone injection may significantly reduce postoperative swelling, pain, and trismus in oral surgery [20]. While we did not observe complications in our three cases, these preventive approaches should be considered when performing high-volume injections in this anatomical region.

While we acknowledge that our technique theoretically carries these risks, several clinical factors may mitigate them. First, the LPM is part of an integrated system with significant functional redundancy provided by other muscles controlling mandibular movement. This functional overlap likely explains why no clinical signs of significant muscle dysfunction were observed in our patients despite the high-volume injection. Second, while our clinical data provides some evidence against significant muscle injury, we must acknowledge several limitations. Postoperative creatine kinase (CK) levels measured on POD 0 were 89 U/L for the first patient, 56 U/L for the second patient, and 86 U/L for the third patient, all within normal reference ranges (76–153 U/L). However, in this context, CK may not be a specific marker for LPM injury, given that SSRO inherently involves trauma to mandibular bone and surrounding muscles, potentially obscuring LPM-specific changes.

Furthermore, our CK measurements were limited to a single time point at POD 0, lacking the temporal resolution to detect delayed elevations that might occur with myotoxicity. These limitations underscore the importance of prioritizing clinical observations such as trismus, mouth opening measurements, and mandibular function, which more directly reflect functional outcomes. Notably, none of our patients developed late trismus or mandibular dysfunction through their discharge dates and follow-up visits, suggesting that despite the potential limitations of our biochemical monitoring, the clinical outcomes remained favorable.

Furthermore, similar anesthesia concepts and approaches providing intramuscular injection techniques in regional anesthesia provide precedent for the safety of intramuscular injections. Palhais et al. demonstrated the safety and efficacy of extrafascial (intramuscular) injection for interscalene brachial plexus block, where 20 mL of ropivacaine was injected into the middle scalene muscle in patients undergoing shoulder surgery without reported instances of muscle necrosis or movement disorders [21]. Similarly, Murouchi et al. reported successful intramuscular quadratus lumborum block use without significant complications. They reported the clinical safety of local anesthetic toxicity in measuring serum local anesthetic concentration compared to the transversus abdominis plane block [22].

For future studies, we propose investigating lower volumes (3–5 mL) in dose–response trials. This reduced volume range was specifically chosen because it approximates or remains within the anatomical volume of the LPM (5–6 cm^3^) and should lower baro/volume trauma while still allowing spread to the pterygomandibular space and pterygopalatine fossa. We hypothesize that if sufficient local anesthetic is administered to fill the LPM, the agent may subsequently spread to the adjacent pterygomandibular space, where it can exert its nerve-blocking effects. Conversely, excessive volumes may not only increase the risk of myotoxicity but also potentially lead to systemic local anesthetic toxicity. Finding this optimal balance—where the volume is sufficient for adequate analgesia but not excessive enough to cause adverse effects—is critical. However, this remains a hypothesis that requires further investigation and validation through additional controlled dose–response studies.

Based on pharmacokinetic data from comparable regional blocks, we can reasonably estimate that bilateral 5 mL injections of 0.25% levobupivacaine (total 25 mg) would likely result in peak plasma concentrations (Cmax) well below 0.5–1.0 µg/mL, which remains substantially below the toxic threshold of 2–4 μg/mL established by Scott and Kopacz and Allen [23, 24]. This Cmax estimate is based on published pharmacokinetic data: thoracic paravertebral blocks with 1 mg/kg levobupivacaine showed Cmax values of 0.48–0.71 µg/mL [25]. In comparison, transversus abdominis plane blocks using 100 mg levobupivacaine resulted in a Cmax of approximately 1 µg/mL [26]. Given that this reduced dose (0.25% levobupivacaine, 5 mL bilaterally for a total of 25 mg) is substantially lower than the doses used in the previously mentioned studies and assuming similar absorption kinetics from the LPM as from other muscle and fascial planes, we anticipate peak plasma concentrations would remain well below toxic thresholds. While this 5 mL volume represents half of our currently reported technique (10 mL per side), potentially balancing safety considerations and clinical efficacy, future dose–response studies are needed to establish the optimal balance between minimizing myotoxicity risk and maintaining adequate analgesic effect. This approach is critical because our technique represents a novel application where comprehensive safety and efficacy data are still being established.

Further investigation with larger patient populations is warranted to validate these initial findings. Our novel lateral pterygoid muscle block technique demonstrated promising clinical efficacy in our three SSRO patients. However, prospective studies with larger patient cohorts are essential to establish this approach’s comprehensive safety profile and long-term therapeutic outcomes. Through such rigorous evaluation, we aim to contribute to advancing regional anesthesia techniques for orthognathic surgery, improving patient comfort and reducing reliance on systemic analgesics.

## Data Availability

The datasets used and analyzed during the current study are available from the corresponding author on reasonable request.
